# Nanoscale studies link amyloid maturity with polyglutamine diseases onset

**DOI:** 10.1038/srep31155

**Published:** 2016-08-08

**Authors:** F. S. Ruggeri, S. Vieweg, U. Cendrowska, G. Longo, A. Chiki, H. A. Lashuel, G. Dietler

**Affiliations:** 1 Laboratory of Physics of Living Matter, École Polytechnique Fédérale de Lausanne (EPFL), 1015 Lausanne, Switzerland; 2 Department of Chemistry, University of Cambridge, CB21EW, United Kingdom; 3 Laboratory of Molecular and Chemical Biology of Neurodegeneration, Brain Mind Institute, École Polytechnique Fédérale de Lausanne (EPFL), 1015 Lausanne, Switzerland; 4 Qatar Biomedical Research Institute (QBRI), Hamad Bin Kahlifa University (HBKU), P.O Box 5825, Doha, Qatar

## Abstract

The presence of expanded poly-glutamine (polyQ) repeats in proteins is directly linked to the pathogenesis of several neurodegenerative diseases, including Huntington’s disease. However, the molecular and structural basis underlying the increased toxicity of aggregates formed by proteins containing expanded polyQ repeats remain poorly understood, in part due to the size and morphological heterogeneity of the aggregates they form *in vitro*. To address this knowledge gap and technical limitations, we investigated the structural, mechanical and morphological properties of fibrillar aggregates at the single molecule and nanometer scale using the first exon of the Huntingtin protein as a model system (Exon1). Our findings demonstrate a direct correlation of the morphological and mechanical properties of Exon1 aggregates with their structural organization at the single aggregate and nanometric scale and provide novel insights into the molecular and structural basis of Huntingtin Exon1 aggregation and toxicity.

The formation of insoluble protein aggregates with cross-β sheet structure, termed amyloids, is implicated in the onset and pathogenesis of several neurodegenerative and systemic human diseases^1^. Within this group, there are nine hereditary polyglutamine (polyQ) diseases, in which a genetic mutation leads to the expansion of the CAG-encoded polyQ tract of the causative protein. When a certain polyQ-length threshold is exceeded, proteins are prone to misfolding, aggregation and formation of β-sheet-rich fibrillar aggregates causing neuronal dysfunction and toxicity^2^,^3^. The proteins involved in the different polyQ diseases lack sequence and structural homology, but they share the common feature of a pathogenic polyQ threshold that determines the disease age of onset and severity^4^,^5^,^6^,^7^.

Huntington’s disease (HD) occurs when the polyQ tract in the first exon (Exon1) of the huntingtin protein (Htt) expands above a length of 36Q^4^,^8^,^9^. Several *in vitro* studies using Exon1 fusion proteins and synthetic polyQ peptides showed a correlation between the polyQ-length and aggregation propensity of Htt^10^,^11^. Therefore, exceeding the pathogenic threshold must alter the structural properties of expanded Huntingtin aggregates conferring toxicity^12^,^13^. Furthermore, several lines of evidence suggest that the first N-terminal 17 amino acids of the Huntingtin protein (Nt17 domain) are a key factor in regulating the subcellular localization, aggregation and toxic properties of the Htt protein and its N-terminal fragments^6^,^14^,^15^. The Nt17 domain dramatically accelerates the polyQ-mediated amyloid aggregation *in vitro*, whereas in a transgenic mice model expressing full-length Huntingtin it prevented nuclear aggregation and reduced HD pathology compared to mice expressing Nt17-truncated Huntingtin^14^,^16^,^17^,^18^. The differential toxicity observed for full-length and Nt17-truncated Huntingtin suggests the existence of intrinsic structural differences between the aggregates formed by these different forms of the protein. Therefore, we hypothesize that there is a correlation between toxicity and individual aggregate biophysical properties modulated by the polyQ content and the Nt17 domain.

Despite increasing evidence linking amyloid formation to human diseases, unraveling the relationship between amyloid formation and toxicity still represents a formidable experimental challenge, mainly because of their nanoscale dimensions and heterogeneous nature. *In vitro* biophysical studies of protein aggregation have been based widely on the use of bulk techniques, such as infrared spectroscopy (IR) and Circular Dichroism (CD)^19^,^20^,^21^. A significant factor, limiting the general applicability of these techniques, is their capability to give only average information on the heterogeneous ensemble of species present in an aggregating amyloid solution, thus precluding a profound investigation of the correlation between the biophysical properties of the individual aggregate species and their toxicity^14^,^19^,^22^,^23^,^24^,^25^. Differently from bulk techniques, single molecule techniques, such as Atomic Force Microscopy (AFM), possess increased robustness in measuring the properties of heterogeneous populations by allowing direct measurements and correlation of the biophysical properties of protein aggregates at the nanoscale and single molecule level.

In this work, we correlate for the first time at the single molecule nanometer scale the morphological, mechanical and secondary structural properties of amyloid aggregates by means of conventional and innovative AFM-based techniques (Fig. 1). We show that infrared nanospectroscopy, coupling AFM and IR spectroscopy (AFM-IR), provides novel insight into the sequence determinants of Exon1 aggregation and the structural basis of Exon1 toxicity through quantitative assessment of the mechanical and structural properties of Exon1 aggregates at the nanoscale^26^,^27^. First, we focused on elucidating the relationship between the polyQ content, the presence of the N17 domain and the biophysical properties of single tag-free Exon1 aggregate species below and above the pathogenic threshold by high-resolution AFM imaging and nanomechanical mapping. Then, we established for the first time the use of AFM-IR exploiting quantum cascade lasers to assess and quantitatively compare the structural organization of oligomeric and fibrillar amyloidogenic species at the single molecule level^28^. Our findings demonstrate a direct correlation of the morphological and mechanical properties of Exon1 aggregates with their structural organization at the single aggregate and nanometric scale, thus providing novel insights into the molecular basis of Exon1 aggregation and toxicity. The results show a clear polyQ-length modulated variation of the biophysical properties of the fibrillar aggregate, indicating that the quality of the intermolecular hydrogen bonds network and the structural organization of the amyloid cross-β sheet structure are fundamental parameters conferring toxicity to the aggregates above the pathogenic threshold.

## The length of the polyQ repeat influences the roughness of Exon1 fibrils

First, we compared the fibrillization capacity of tag-free Exon1 and Nt17-truncated Exon1 with polyQ-lengths below (22Q) and above the pathogenic threshold (42Q) by conventional Atomic Force Microscopy (AFM). The AFM morphology maps, in Fig. 2, show that for both proteins, full length and Nt17-truncated, fibrillation occurs both below and above the pathogenic threshold (>36Q), consistently with previous studies^2^,^12^,^19^,^29^. Indeed, all samples showed the formation of fibrils with height of approximately 6–10 nm, typical of amyloid structures, which were more abundant in the case of expanded proteins (Fig. 2a,b). Then, we assessed the conformational change of proteins over time by means of Circular Dichroism (CD) (Fig. 2c,d). At 0 hours, prior to aggregation, all the proteins retained a predominantly disordered structure showing a minimum at 205 nm in their CD spectra, identical to the secondary structure previously reported for monomeric tag-free Exon1 proteins produced by recombinant protein expression, semisynthesis or solid phase peptide synthesis^12^,^13^,^30^. For pure polyQ peptides, the minimum is usually observed at 200 nm^31^. However, fusing proline-rich sequences to polyQ peptides was shown to cause a red-shift in the CD spectra of polyQ peptides^32^. In the case of Exon1 it is likely that the transient α-helical structure of the Nt17 domain also contributes to the red-shift of the CD spectra^33^,^34^. After 3 days at 37C, although we could detect the formation of fibrils with cross-sectional dimensions typical of amyloids by AFM, a conformational shift towards the cross-β structure in the CD spectra was absent for unexpanded proteins, suggesting that the majority of the protein remains monomeric and disordered. For the proteins containing polyQ repeats above the pathogenic threshold, the spectra showed a clear conformational shift towards 215 nm, indicating the formation of amyloid fibrils with cross-β sheet structure. This suggests a structural difference between aggregates by Exon1 proteins with polyQ repeats below and above the pathogenic threshold. Consistent with previous studies, the removal of the Nt17 domain enhanced the aggregation of both Exon1-22Q and Exon1-42Q. The absence of a change in the CD signal despite the presence of fibril aggregates suggest that they lack β-sheet structure and/or their structural properties are masked by the CD signal from the soluble proteins. Therefore, we initially sought to investigate the structural properties of the aggregates formed by Exon1-22Q and Exon1-42Q by measuring their morphological and mechanical properties at the single aggregate level. To investigate the role of polyQ-repeat length in modulating the biophysical properties of the Exon1 aggregates, we studied proteins with variable polyQ repeat lengths ranging from 14Q–42Q. High-resolution AFM imaging allowed determination of the profile roughness of single amyloid fibrils as a function of polyQ repeat length with sub-nanometer resolution (Fig. 2e,f and Supplementary Figs 1–5). These measurements demonstrated that the profile of both Nt17-truncated and Exon1 fibrils became smoother as a function of the increasing polyQ content (Fig. 2g,h). Notably, fibrils below the pathogenic threshold could not reach a degree of smoothness similar to that of fibrils formed by proteins with expanded polyQ tract, indicating that the order of the β-sheet structure depends on fibril maturity and polyQ content. Finally, a linear fit of the roughness as a function of the polyQ content showed a stronger dependence for the Nt17-truncated Exon1 proteins (Fig. 2g,h).

## PolyQ content determines fibril stiffness

We previously reported that an increase of cross-β sheet content and an improved hydrogen bonding network in amyloid aggregates are positively correlated with an increase of their intrinsic stiffness^35^. Here, the AFM-based fast force-volume system (Quantitative Imaging - QI)^25^ was utilized to investigate the nanomechanical properties of the fibrillar structures as a function of the polyQ repeat length for both Nt17-truncated and Exon1 proteins (Fig. 3).

We measured the nanomechanical properties (Fig. 3a–d and Supplementary Figs 6 and 7) for both Nt17-truncated and Exon1 proteins with unexpanded and expanded polyQ tracts after 3 days incubation since all proteins showed fibril formation at this time point. In addition to fibrils, spherical oligomers were observed in all cases. They were the predominant species formed by proteins with polyQ repeats below 22Q and their presence decreased with increasing polyQ repeat length. First, as reference, we measured the intrinsic stiffness of the spheroidal oligomeric structures present in the morphology maps. In the case of Exon1 with 14Q, the spheroidal species had a stiffness of 0.98 ± 0.38 GPa, while in the case of Nt17-truncated proteins with 22Q, oligomeric aggregates possessed a Young’s modulus of 1.56 ± 0.42 GPa (Fig. 3e,f). Then, we investigated the mechanical properties of the elongated fibrillar structures. Fibrils formed by Exon1 proteins with a polyQ repeat length of 14Q had a stiffness of 1.5 ± 0.7 GPa (Fig. 3b). This value was significantly higher compared to oligomers in the same sample and it was consistent with the stiffness reported in our previous work for protofibrillar *non-mature* amyloid structures formed by α-synuclein, aβ-42 and Josephin of ataxin-3^35^,^36^. Fibrils with a polyQ-repeat length of 22Q had a stiffness of 1.72 ± 0.79 GPa (Nt17-truncated) and 2.03 ± 0.77 GPa (Exon1) indicating that those fibrils do not possess a fully mature cross-β structure stabilized by intermolecular hydrogen bonds. Finally, the measurements demonstrated that the Young’s modulus of fibrillar structures increases as a function of the polyQ-length up to a value of ≈2.7–2.8 GPa for fibrils with a polyQ length of 42Q (Fig. 3b–d). These values provide evidence that fibril stiffness directly correlates with polyQ content and values for fibrils above the pathogenic threshold were fully consistent with the generally accepted values of the stiffness of a mature and complete amyloid cross-β sheet structure stabilized by a tight network of hydrogen bonds^36^,^37^,^38^,^39^,^40^.

## AFM-IR Structural Characterization

In the course of the aggregation, CD measurements showed that the secondary structure of Nt17-truncated and Exon1 proteins with a polyQ-length above the pathogenic threshold shifted towards β-sheet structure, which is generally associated with amyloids formation. On the contrary, proteins with polyQ-lengths below the pathogenic threshold (<36Q) seemed to retain their structure, even-though we observed significant fibril formation by AFM. To investigate further the structural differences between the aggregates formed by the unexpanded and expanded polyQ proteins, we exploited, for the first time, infrared nanospectroscopy to investigate and compare the secondary and quaternary structure of oligomers and fibrils at the single aggregate level.

We deposited Exon 1 samples on freshly cleaved gold substrates (Supplementary Fig. 8) and used a gold-coated tip in order to reach single amyloid fibril resolution by near-field electromagnetic field trapping^28^. Infrared nanospectroscopy enabled the mapping, together with topography of the IR absorption of the sample (Supplementary Fig. 8)^41^,^42^. After a morphology map was completed, we could point the AFM tip on the top of a single fibrillar species and acquire its IR absorption spectra (Supplementary Figs 9 and 10). In particular, we investigated IR absorption in the range of amide band I and II of proteins. Notably, amide band I absorption derives mainly from backbone C=O stretching vibrations with frequencies at 1700–1600 cm^−1^ and the exact band position and shape is determined by the backbone conformation and by the secondary structure of the aggregate under investigation^43^,^44^.

First, we studied the secondary structure content of amorphous and rod-like oligomeric aggregates of unexpanded Exon1 proteins (22Q) (Fig. 4a,b). In the case of oligomeric structures (22Q), the IR absorption spectra and their second derivative presented a strong absorption at 1660 cm^−1^ (Fig. 4c,d and Supplementary Fig. 11), consistent with previous studies^19^. This was ascribed to an overlap of the side chain absorption of the highly abundant glutamine residues (≈1670–1660 cm^−1^) with the α-helical secondary structure conformation of the Nt17 domain (1654–58 cm^−1^)^45^. The oligomeric structures presented a weak structural contribution related to antiparallel β-sheet content (1692 cm^−1^). Second, we investigated the fibrillar structures of unexpanded Exon1 proteins (22Q) (Fig. 4e,f,). The IR spectra acquired on these species presented enhanced absorption at 1692 cm^−1^, which correspond to antiparallel β-sheet structure and a new broad component of absorption at about 1645–1635 cm^−1^, which was ascribed to the convolution of random coil (≈1645 cm^−1^) and the early formation of high-density amyloid β-sheet (≈1635 cm^−1^) conformations (Fig. 4c,d). The comparison of the spectroscopic responses of the two different amyloidogenic species showed a chemical shift of the main component from 1658 cm^−1^ for the oligomers to 1663 cm^−1^ for the fibrils. This indicated that oligomeric structures possess a relative higher α-helical content compared to fibrillar aggregates, which exhibited the β-sheet spectroscopic signature of amyloids.

Next, we examined the spectroscopic properties of fibrils formed by expanded Exon1 (42Q) (Fig. 4g,h). The amide band I absorption contained three main contributions deriving from: amyloid β-sheet secondary and quaternary structures (1635 cm^−1^), the superimposition of signal due to α-helix conformation (1658 cm^−1^) and glutamine side chain vibrations and β-turns (1684 cm^−1^). The comparison of the spectroscopic response of fibrils below and above the pathogenic threshold showed that the expanded Exon1 fibrils have an increased β-sheet structure content and β-turn conformation compared to unexpanded Exon1 proteins, indicating the formation of a compact and mature amyloid structure. Finally, we assessed the IR absorption and second derivative spectra of Nt17-truncated Exon1 aggregates below (22Q) and above the pathogenic threshold (42Q) (Fig. 4i–l). Similar to Exon1 proteins, we could observe a shift of the Amide band I towards the wavenumbers that correspond to a higher content of amyloid β-sheets structure for expanded Nt17-truncated Exon1 proteins. However, we could still observe a shoulder band that corresponds to random coil conformation.

Notably, high cross-β sheet content directly correlates with the organization of the amyloid structure and order of its intermolecular hydrogen bonding network. The improvement of this network, in number of bonds and order, is reflected in the shift of the main component of the spectrum from 1663 cm^−1^ for the unexpanded proteins (22Q) to 1659 cm^−1^ for the expanded ones (36/42Q). Indeed, it has been reported that glutamine side chains IR absorption are sensitive to hydrogen bonding, normally causing a shift of the IR spectrum towards lower wavenumbers^45^,^46^. This shift was consistently observed for the aggregates of both Nt17-truncated and Exon1 proteins.

The analysis of the second derivative spectra showed a clear difference between fibrils formed from Nt17-truncated and Exon1 proteins. Below the pathogenic threshold, their secondary structure was similar. However, above the pathogenic threshold Exon1 proteins showed high β-turn and β-sheet structural content, while Nt17-truncated Exon1 aggregates showed antiparallel β-sheet structure and a small contribution from random coil conformation.

## Discussion

There is consensus that Htt aggregation plays an important role in the pathogenesis of HD, however the nature of the toxic species remains elusive. There is contrasting evidence in favor of a protective or a toxic role of the protein inclusions in HD pathogenesis^8^,^47^,^48^,^49^,^50^. Numerous recent HD studies support a discrepancy between Htt aggregation and toxicity showing that inclusion formation in striatal neurons by mutant Htt can be protective or that Exon1 with polyQ repeats below the pathogenic threshold can still aggregate *in vitro* and form *bona fide* amyloid-like fibrils^12^,^51^,^52^,^53^. The majority of published studies are based on correlating biophysical characteristics that are measured using bulk techniques on samples that contain heterogeneous mixtures of Htt aggregates. These techniques do not allow one to account for the heterogeneity of Htt aggregates and/or elucidate the relative contribution of different aggregates in these mixtures. We hypothesize that the observed differences in the toxicity mechanism may be reconciled by taking into account the structural diversity of oligomeric and fibrillar aggregates formed by the proteins with polyQ-lengths below or above the pathogenic threshold^50^. Therefore, we sought to exploit experimental approaches that allow assessment and comparison of the ultrastructural properties of individual aggregate species in complex mixtures. We addressed the current limitations of bulk techniques by using high-resolution AFM imaging, a fast force-volume system (Quantitative-Imaging - QI) and employing for the first time in literature, AFM-IR exploiting quantum cascade lasers (QCL). These AFM-based single molecules techniques enabled detecting changes in the morphological, mechanical and structural properties of single oligomeric and fibrillar species. The results we obtained demonstrated that the smoothness and ordering of Exon1 and Nt17-truncated Exon1 fibrils increased as a function of polyQ-length, suggesting a higher structural order in the cross-β sheet network. Next, we confirmed the improved organization and order of the β-sheet structure by measuring the nanomechanical properties of the aggregates. The reported measurements of the Young’s modulus of the fibrillar aggregates showed that their intrinsic stiffness proportionally correlates with the polyQ content. We previously demonstrated that the cross-β sheet content, with its tight network of hydrogen bonds, is the main factor determining the stiffness of the amyloidogenic structure. Thus, the linear increase of the intrinsic stiffness indicates an improvement of the order of the amyloidogenic cross-β sheet structure and more specifically, of the number of intermolecular hydrogen bonds involved in the formation of this network. Finally, we could investigate the secondary and quaternary organization of single aggregates of unexpanded (22Q) and expanded (42Q) Nt17-truncated and Exon1 proteins by AFM-IR. Our aim was to determine quantitatively the structural difference in their cross-β sheet structure. We were able to demonstrate that Exon1 oligomers (22Q) lacked ordered β-sheet structure and were dominated by an α–helical conformation and glutamine signature originating from the Nt17 domain and the polyQ-rich sequence, respectively. On the contrary, fibrillar structures were rich in amyloidogenic cross-β sheet structure. The amyloidogenic content increased as a function of polyQ-length. Interestingly, Exon1 aggregates exhibited absorption at 1684 cm^−1^ characteristic for β–turn, while this structural feature is absent in Nt17-truncated Exon1 aggregates.

In this work, as modeled in Fig. 5, we present evidence from three independent high-resolution measurements that the polyQ content determines a deep difference in the ordering of the cross-β sheet structure of Huntingtin Exon 1 aggregates. This change in aggregates ultrastructure correlates with the crossing of the pathogenic threshold and therefore it could potentially be linked with the toxicity mechanism. Furthermore, we were able to detect a distinct structural signature of the β-sheet network in Nt17-truncated (antiparallel β-sheet) and Exon1 aggregates (β–turn). Our results suggest that in addition to playing an important role in accelerating the aggregation of Exon1, the Nt17 domain seem to eliminate also the antiparallel organization for the expanded Exon1 fibrillar aggregates. This structure is substituted by a compact β-turn conformation showing a lower morphology dependence (roughness) on the polyQ content. The importance of this finding is highlighted by recent findings from Gu *et al*. showing that the expression of Nt17-truncated mutant Htt in BAC transgenic mice causes a more severe disease-like phenotype compared to the full-length Huntingtin protein^17^. This behavior could be related to a higher templating capacity of the highly organized purely antiparallel cross-β structure of Nt17-truncated proteins above the pathogenic threshold. The structural information provided here could be central to develop a general structural-based pharmacological approach to *polyglutamine diseases*^3^,^4^,^7^,^54^.

In general, the molecular origin and mechanistic link between amyloid formation and disease aetiology remain unclear and no disease modifying therapies are available for these disorders. We believe that the development of new biophysical methodologies, which combine the investigation of morphological and ultrastructural properties of amyloid at the nanoscale, represents a fruitful avenue to address the challenge of unraveling protein self-assembly, monomer misfolding, amyloid polymorphism and formation.

## Materials and Methods

### Aggregation assays

Nt17-truncated Huntingtin Exon1 was produced by recombinant protein expression, while Huntingtin Exon1 (Exon1) was generated from those fragments by subsequent Semisynthesis (*Semisynthesis of mutant (43Q) Httex1 enables investigation of the crosstalk between N-terminal phosphorylation and acetylation in regulating the aggregation of exon1 of the Huntingtin protein.* Anass Chiki, Sean M. De Guire, Francesco S. Ruggeri, Sophie Vieweg, Ritwik Burai, Urszula Cendrowska, Bruno Fauvet, Giovanni Dietler and Hilal A. Lashuel)^12^. Nt17-truncated Exon1 (Htt18-90(Q18C)-14/22/28/36/42Q) was expressed in fusion with the Ssp DnaB intein in ER2566 *E. coli* (New England Biolabs (NEB), # E6901S) using the pTWIN1 vector (NEB, # N6951S). The fusion protein was purified via Ni-NTA affinity purification and spliced afterwards at pH 7.0 and at room temperature for 2–3 h. The splicing product Nt17-truncated Exon1 was finally purified by preparative C4 reverse-phase high performance liquid chromatography (RP HPLC) and lyophilized. To obtain Exon1, recombinant Nt17-truncated Exon1 was ligated with a synthetic C-terminal peptide thioester comprised of amino acids 2–17 of Exon1 by Native Chemical Ligation (NCL)^55^. During the NCL the C-terminal peptide thioester reacts with the N-terminal cysteine of the recombinant protein forming a new thioester (transesterification). The ligated product undergoes an irreversible S-N-acylshift generating a stable amide bond and thus, Exon1. To remove the cysteine at the ligation site, the ligation product was desulfurized leaving behind an alanine at position 18 (Q18A mutation). The final product was purified by preparative C4 RP-HPLC and lyophilized.

The aggregation of was performed from lyophilized proteins, which were disaggregated by addition of pure trifluoroacetic acid. When acid was evaporated, proteins were dissolved in 10 mM PBS. The pH was adjusted to 7.2–7.4 and protein solutions were filtered through 100 kDa centrifugal filter units (Millipore, #MRCF0R100 to remove pre-formed aggregates. Nt17-truncated and Exon1 proteins with variable polyglutamine polyQ tract length (14, 22, 28, 42 Q) were incubated at 37 °C at concentrations of 7–9 μM for unexpanded Exon1 (6-28Q), 8 μM for Nt17-truncated Exon1 36Q/42Q and 4 μM for expanded Exon1 36/42Q. An aliquot of each sample for experimental measurements was collected at several times point between 0 hours and 14 days.

### Circular Dichroism

Samples were analyzed at room temperature (RT) using a Jasco J-815 CD spectrometer. An average of 5 spectra per sample was collected in the range of 190−250 nm using a 1.0-mm-optical-pathlength quartz cuvette. Data points were acquired every 0.2 nm in the continuous scanning mode at a speed of 50 nm/min with a digital integration time of 2 s and a bandwidth of 1 nm. Processed spectra were obtained by subtracting the baseline signal due to the buffer and cell contribution from the protein spectra, and by smoothing using a binomial filter with a convolution width of 99 data points.

### Atomic Force Microscopy and nanoIR samples preparation

Analysis by conventional Atomic Force Microscopy and nanoIR2 (Anasys Instrument, USA) was performed on positive gold surfaces (Platypus Technologies, USA) and on positively functionalized mica substrates. In the first case, an aliquot of 10 μl of each sample was deposited on the freshly stripped gold for 10 minutes. Successively, the droplet was rinsed by 1 ml of Milli-Q water and dried by a gentle stream of nitrogen gas. In the latter case, we cleaved the mica surface and we incubated it for 1 minute with 10 μl of 0.5% (v/v) (3-Aminopropyl)triethoxysilane (APTES) in Milli-Q water. Then, the substrate was rinsed three times with 1 ml of Milli-Q water and dried by gentle stream of nitrogen gas. Finally, for each sample, an aliquot of 10 μl of the solution was deposited on the positively functionalized surface. The droplet was incubated for 10 minutes, then rinsed by 1 ml of Milli-Q water and dried by the gentle stream of nitrogen gas. In both cases, preparation was carried out at room temperature.

### Conventional Atomic Force Microscopy measurements

High-resolution images (1024 × 1024 pixels) were collected using an NX10 Atomic Force Microscopy (Park Systems, South Korea) in ambient conditions and in non-contact Amplitude Modulation (NC-AM). We imaged square areas of 2 × 2 μm^2^ and 4 × 4 μm^2^. We performed all the measurements using ultra-sharp cantilevers (SSS-NCHR, Park Systems, South Korea) with resonance frequency of 330 kHz and typical radius of curvature of 2 nm.

In AM-AFM, the tip is excited with fixed amplitude by an external force. The tip’s interaction with the sample changes its motion and causes a difference between the initial and final tip amplitude, which results in a phase shift. Such phase changes reflects the dissipated energy during sample-tip interaction. By recording the phase difference, AM-AFM phase images were created contemporary to morphology maps. High phase changes represent a strong tip-sample interaction and strong interaction force can lead to sample deformation by the tip, while low phase change represent weak tip-sample interaction. In order to compare consistently the height of different samples, we established standardized experimental scanning conditions and we maintained a regime of phase change in the order of ≈Δ20° (Supplementary Fig. 1).

Raw images were flattened with the XEI software (Park System, South Korea). In order to keep consistency in the further statistical analysis, all images were processed with the same parameters. First, images were flattened by a plane and then line by line at a 1^st^ regression order. This second step was repeated until the flat baseline in line profile of the image was reached (Supplementary Fig. 3). In case of very crowded images or images with exceptionally high aggregates, we flattened with the line scope at a 2^nd^ regression order in the second step. During the process of flattening of the images, the aggregates were masked from the calculation to avoid a modification and underestimation of their height.

We compared the structural difference between different samples through an accurate control of the sample-tip interaction during scanning the probe through the sample (Materials and Methods and Supplementary Fig. 1). Furthermore, before analyzing the fibrillar structures and their roughness, the images were flattened through a standardized process and the surface roughness was accurately measured (Supplementary Figs 2 and 3). To perform the analysis, the maximum height profile of single fibrillar structures was traced. Then, we normalized the profile and studied its roughness (Fig. 2e,f and Supplementary Fig. 4). To quantify the fibrillar roughness, we represented this parameter as the standard deviation of the histogram representing the deviation of the fibril profile from its average height (Fig. 2e,f).

### Cross-sectional dimensions statistical analysis of fibrillar species

We performed a statistical analysis on the cross-sectional dimensions of individual fibrillar structures. We traced their average height and length by means of the home-built DNA trace software^56^. The software allows tracing the section along each structure by the linear connection of its highest points. Fibrils were traced with a constant step of 4 nm (2 pixels for the 4 × 4 μm^2^ images and 4 pixels between two points for the 2 × 2 μm^2^ images) (Supplementary Fig. 4). Notably, we chose a step ideally equal to the diameter of the tip we used to retrieve the morphology of the sample. The average height of each structure was calculated as the average of the heights of all tracing points, while the length of the fibrils was calculated as the number of points used to trace the molecule multiplied for the step size (Supplementary Fig. 4).

In order to collect statistical properties of individual fibrils, we excluded fibrils crossing with each other or fibrils assembled in aggregates. The ensemble of all measurements was represented by a histogram (Supplementary Fig. 4). Data were analyzed and histograms were created using OriginPro (OriginLab) software.

### Statistical analysis of the roughness of fibrillar species

After tracing the fibrils, we analyzed their profile roughness. To calculate this parameter, we normalized each profile by subtracting its average height (Supplementary Fig. 4). In such way, we obtained a distribution of the profile below and above the zero, which we represented in a histogram (Supplementary Fig. 4). In order to avoid artifacts due to the short length of fibrils in some samples, we calculated this quantity just for fibrils with minimal length of 60 nm, excluding the regions close the fibril ends.

For each sample, we added the roughness distribution of several fibrils, in order to sum up an overall minimal total length of at least 2 μm. To estimate the average roughness of the fibrillar structures of each sample, the histogram was fitted by a Gaussian curve, whose standard deviation represents their average roughness. All the Gaussian fits reached statistical significance with a *p-value* < 0.001. We calculated the experimental error on the roughness as the sum of the standard deviation, the average roughness of the sample (Supplementary Fig. 2) and the electrical noise of the AFM (≈0.03 nm). Data were analyzed and histograms were created by means of OriginPro (OriginLab) software.

### Quantitative imaging measurements

The nanomechanical measurements were obtained by using a JPK Nanowizard III atomic force microscope (JPK Instruments AG, Germany). We chose CSC cantilevers (μMasch, Spain), with a nominal spring constant of 1.00 N/m and a maximum apical radius of 20 nm. Indeed, the choice of the tip by one side depends on the need to be sensitive to the expected stiffness of the samples, but on the other on the need to reduce the damage of the molecules. Before each experiment, we calibrated the mechanical properties of the tip using the JPK software^57^. All images were collected by means of the quantitative imaging (QI) modality, an evolution of the force-volume mode in which the AFM tip is placed in fast oscillation over the sample and the deformation of the cantilever is recorded to reconstruct an image formed by a large number of force distance (FD) curves. In this fast modality, the AFM can perform several FD curves without requiring the tip to oscillate at a resonant frequency, thus allowing the use of the ideal tips for the stiffness investigation process. All the presented images contain up to 256 × 256 pixels and on each pixel we collected a 2048 points FD curve. The length of the curves was 80 nm and the maximum imaging speed was 3 lines per second. The tip-sample interaction was limited to a maximum cantilever deflection of 5 nm (i.e. 5 nN). We imaged at least 3 different areas per sample. In total, the average values were calculated over more than 100 individual protein aggregates. Depending on the size and on the resolution of the image, the stiffness of each molecule was measured on a minimum of 20 points. Processing was done in a semi-automated way with the JPK data processing software, assuming that the cantilever behaved accordingly to the Hooke law (i.e., the deflection of the cantilever is directly proportional to the vertical component of the force applied on the tip). In this case, the FD curves collected on the sample can be subtracted from the FD curves obtained on a hard glass substrate, resulting in indentation curves (Supplementary Fig. 7). The shape of each indentation curve was used to calculate the mechanical properties of the sample and specifically to measure its intrinsic Young’s modulus. In detail, we retrieved the elastic modulus fitting the indentation curves using the Hertz model^58^. We assumed a Poisson’s ratio of the aggregates of 0.3^57^. Measures of the average stiffness values for each sample (monomers, oligomers, fibrils) are expressed in giga Pascal (=10^9^ Pa) (GPa) as the mean ± standard deviation (SD).

### NanoIR measurements

**Figure 1:**
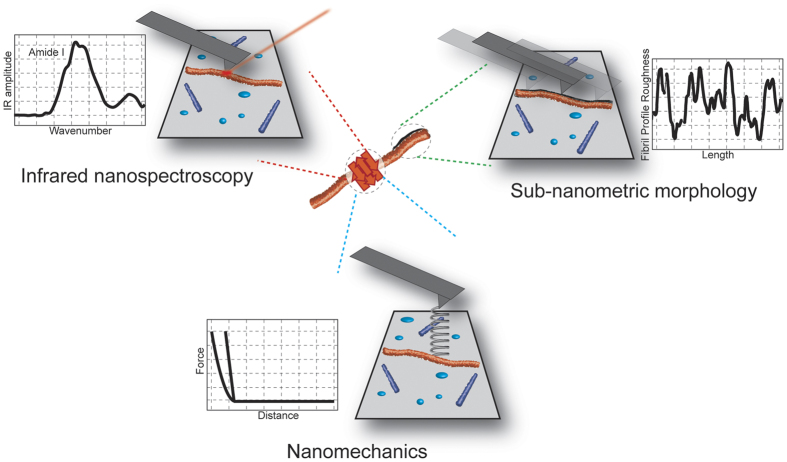
Ultrastructural characterization of single amyloid aggregates. (Top-left) Secondary structure investigation by means of Infrared nanospectroscopy. (Top-right) Sub-nanometric characterization of fibrils roughness by high-resolution AFM microscopy. (Bottom) Nanomechanical measurements of aggregates Young’s modulus.

**Figure 2:**
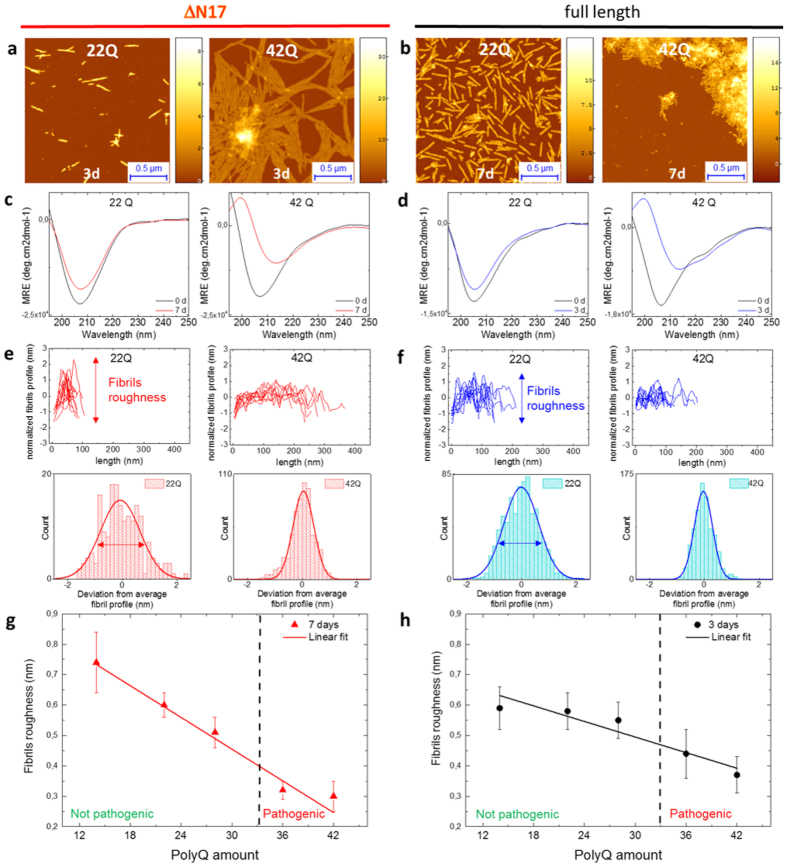
Morphological properties of fibrils. (**a,b**) AFM maps, (**c,d**) Circular dichroism measurements before incubation (0 days) and after incubation (3,7 days), (**e,f**) fibrillar roughness analysis of Nt17-truncated and full length proteins with 22Q and 42Q. Roughness of fibrillar structures as a function of polyQ stretch length of (**g**) Nt17-truncated at 7 days and (**h**) full-length proteins after 3 days incubation. Lines represent the linear fit of the roughness as a function of the polyQ content.

**Figure 3:**
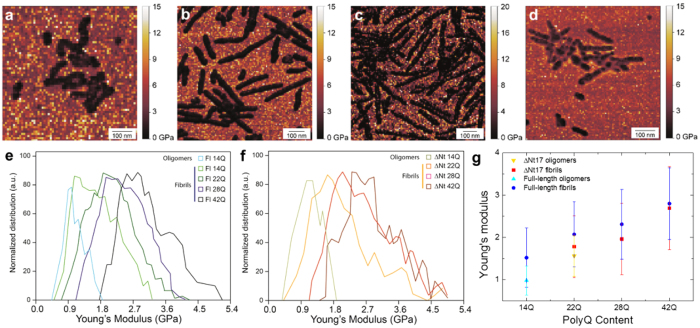
Nanomechanical measurements of fibrils stiffness on mica. QI stiffness maps for Exon1 proteins with polyQ stretch length of (**a**) 14Q, (**b**) 22Q, (**c**) 28Q and (**d**) 42Q at 3 days of incubation. (**e**) Stiffness distribution of Exon1 fibrillar structures. (**f**) Stiffness distribution of Exon1 fibrillar structures Nt17-truncated at 3 days of incubation. (**g**) Young’s modulus increase as a function of polyQ stretch length.

**Figure 4:**
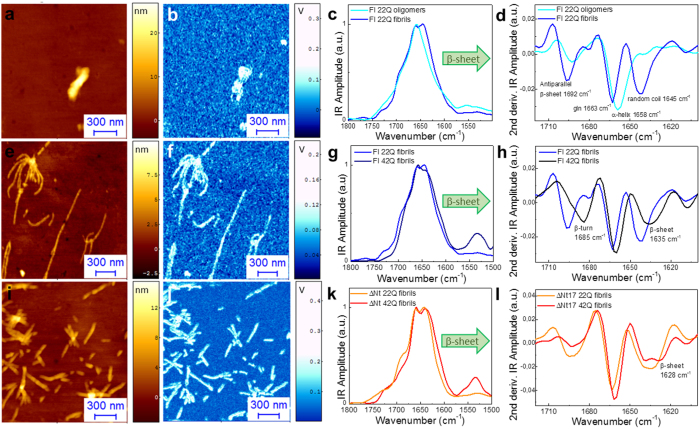
AFM-IR of Exon1 aggregates. (**a**) AFM morphology and (**b**) IR absorption maps of oligomers with 22Q. (**c**) IR spectra and (**d**) second derivatives of Exon1 oligomers with 22Q. (**e**) AFM morphology and (**f**) IR absorption maps of Exon1 fibrils with 22Q. (**g**) IR spectra and (**h**) their second derivatives of oligomers and fibrils of Exon1 with 22Q. (**i**) AFM morphology and (**j**) IR absorption maps of Exon1 fibrils with 42Q. (**k**) IR spectra and (**h**) the second derivatives of Exon1 fibrils with 22Q and 42Q. (**l**) IR spectra pand (n) second derivatives of Nt17-truncated aggregates with 22Q and 42Q.

**Figure 5:**
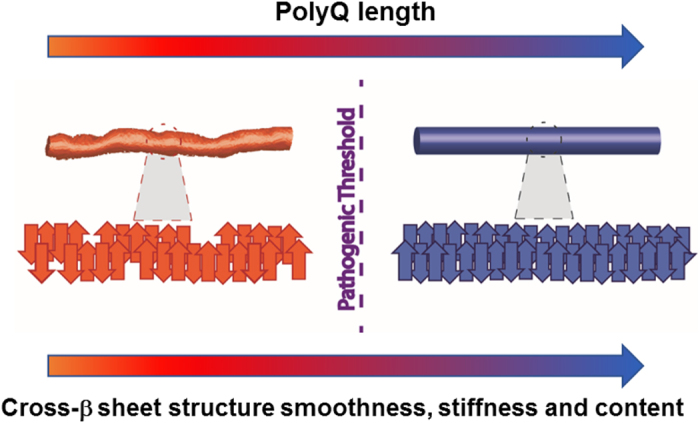
PolyQ content determines quality of the amyloidogenic cross-β sheet structure.

For all the nanoscale IR measurements, we used a nanoIR2 platform (Anasys, USA), which combines high resolution and low noise AFM with a tunable IR Quantum Cascade laser (QCL). Compared to the conventional nanoIR system, the pulsing of the QCL laser can be tuned at the cantilever’s resonance frequency and the IR electromagnetic field can be trapped in the nanogap between a sharp gold-coated AFM tip and a gold-coated substrate. The higher apical radius of the gold-coated tips, 10-fold more than the ultra-sharp tips used to acquire the morphology maps in Fig. 1, causes an apparent broadening of the observed structures. However, the electromagnetic trapping enhances the instrument spectral resolution^28^. Tip-enhanced infrared nanospectroscopy allows nanoscale measurements of IR absorption as a function of the wavenumber to characterize specimens at spatial resolutions not previously achievable and allowing detecting objects with thickness of ≈2 nm and with a lateral resolution as small as the radius of the AFM tip. This minimal measurable thickness is defined by the minimal detectable photo-thermal expansion of the sample (Photothermal Induced Resonance Effect, PTIR). On the other hand, the lateral resolution of an isolated object is only limited by the sharpness of the AFM lever and by thermal diffusion to ≈25 nm.

Samples were deposited on freshly cleaved gold-coated surfaces. We scanned the samples by the nanoIR microscopy system, with a rate line within 0.1–0.2 Hz and in contact mode. We used a silicon gold coated PR-EX-nIR2 (Anasys, USA) cantilever with a nominal radius of 30 nm and an elastic constant of about 0.2 N/m. All images have a resolution of at least 512 × 150 pixels per line. The AFM images were treated using SPIP software. The height and quantum cascade laser (QCL) resonance peak amplitude images were first order flattened.

The spectra were collected by placing the AFM tip on the top of the structure under investigation with a laser wavelength sampling of 2 cm^−1^ with a spectral resolution of 0.1 cm^−1^ and 256 co-averages, within the range 1200–1800 cm^−1^. In order to have statistically relevant data, several spectra were acquired on the top of several amyloidogenic structures. At each position, the spectrum was obtained as the average of 5–20 spectra and by subtracting the baseline signal of the gold substrate (Supplementary Fig. 9). Successively, they were smoothed with a Savitzky-Golay filter (2^nd^ order, 9 points). The second derivative of the IR absorption in the amide band I region allowed for de-convolution of the main contributions to the absorption due to the vibrations of different secondary structure conformations.

Spectra were analysed using the microscope’s built-in Analysis Studio (Anasys) and OriginPRO. All measurements were performed at room temperature and with laser power between 7–12% of the maximal one.

## Additional Information

**How to cite this article**: Ruggeri, F. S. *et al*. Nanoscale studies link amyloid maturity with polyglutamine diseases onset. *Sci. Rep.* **6**, 31155; doi: 10.1038/srep31155 (2016).

## Supplementary Material

Supplementary Information
