# Supplementary material for: Nanoscale studies link amyloid maturity with polyglutamine diseases onset
Source: Sci Rep. 2016 Aug 8;6:31155. doi: 10.1038/srep31155 (PMC4976327; doi:10.1038/srep31155)
Supplement: Supplementary Information [file srep31155-s1.pdf]

Nanoscale studies link amyloid maturity with polyglutamine diseases onset

F.S. Ruggeri[1,2*], S. Vieweg, U.[3*] Cendrowska[1,3], G. Longo[1], A. Chiki[3], H. A. Lashuel[3,4+], G. Dietler[1+]

[1] Laboratory of Physics of Living Matter, École Polytechnique Fédérale de Lausanne (EPFL), 1015 Lausanne, Switzerland.

[2] Department of Chemistry, University of Cambridge, CB21EW, United Kingdom

[3] Laboratory of Molecular and Chemical Biology of Neurodegeneration, Brain Mind Institute, École Polytechnique Fédérale de Lausanne (EPFL), 1015 Lausanne, Switzerland, and [4]Qatar Biomedical Research Institute (QBRI), Hamad Bin Kahlifa University (HBKU), P.O Box 5825, Doha, Qatar.

* These authors contributed equally to this work.

Corresponding authors: H. A. Lashuel[2+], G. Dietler[1+]

SUPPLEMENTARY INFORMATION

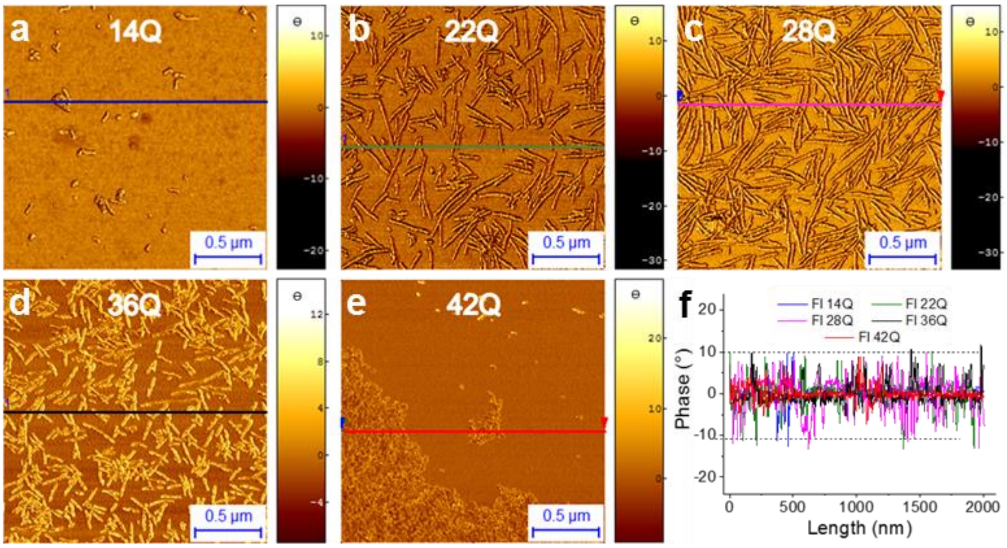

Supplementary Fig. 1. Tip-sample interaction control. a-e) AFM phase images of full-length exon1 structures with different polyQ tract length after 3 days of incubation. f) Graph representing phase change in each image, which was always smaller than Δ20°.

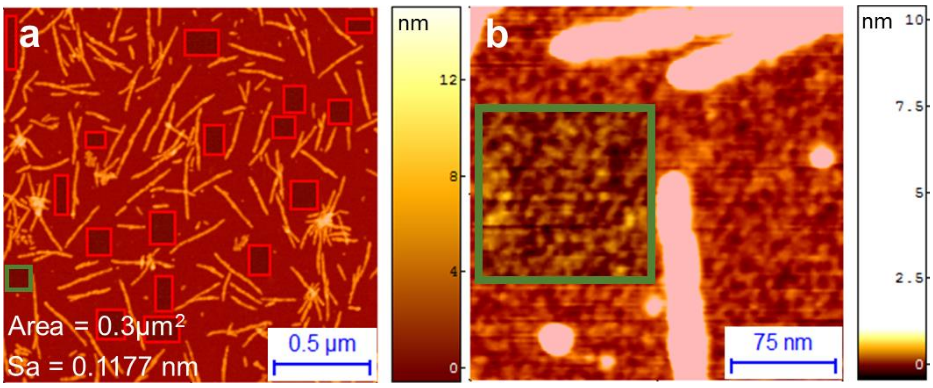

Supplementary Fig. 2. Measurement of surface roughness. a) AFM image with chosen areas from where the surface roughness (Sa) was extrapolated. b) Detail view on one of the areas for roughness calculation.

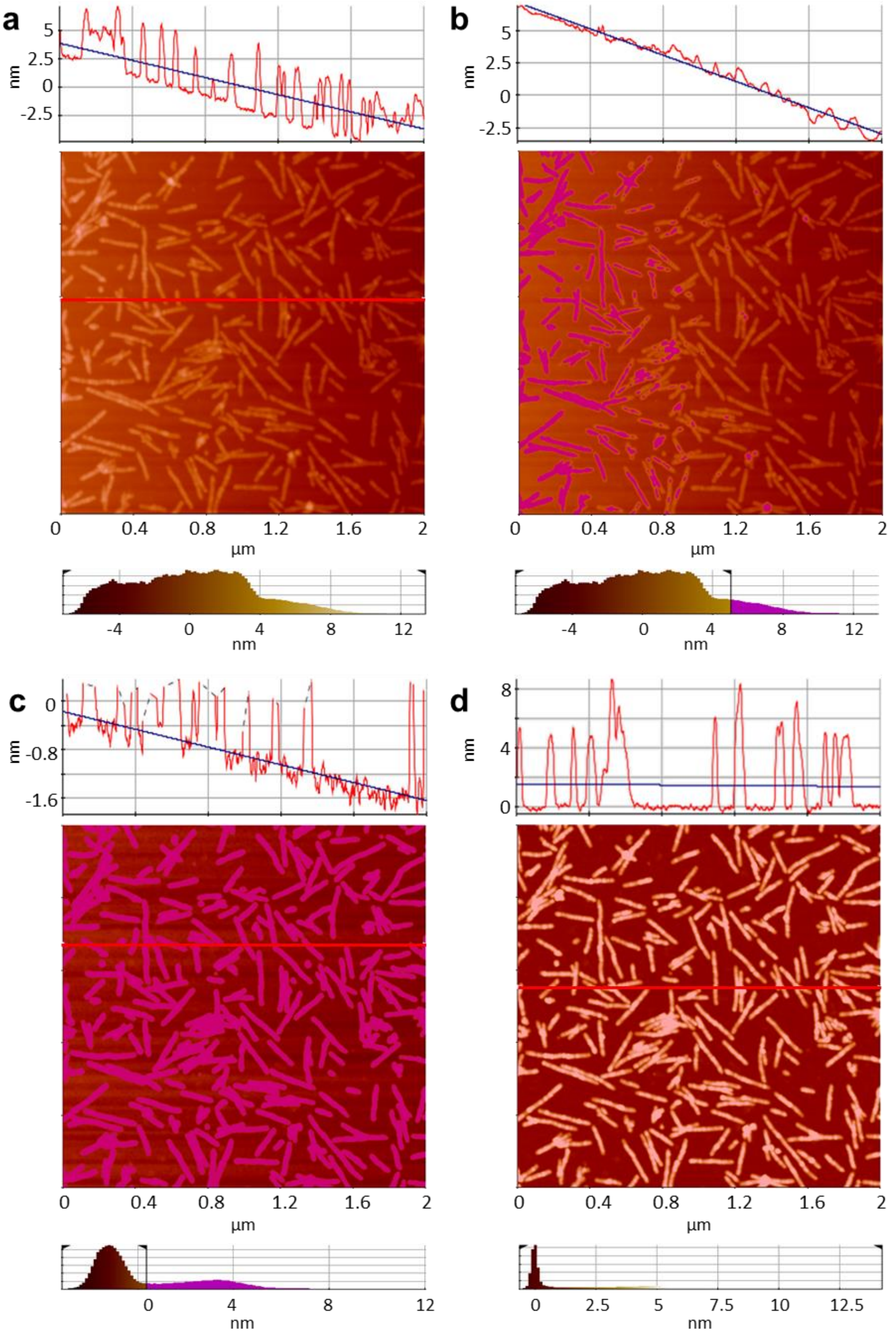

Supplementary Fig. 3. Process of flattening of the AFM images. Top of each panel shows a profile of the sample surface (red line) and its height. Middle panel shows the corresponding AFM image and the lower panel shows a histogram of the height of all pixels in the image. a) Raw AFM image without any treatment. b) Image treated with whole plane flattening, fibrillar structures (pink color) are masked from the surface subtraction. c) Image processed with line-by-line flattening subtraction. D) Final flattened image.

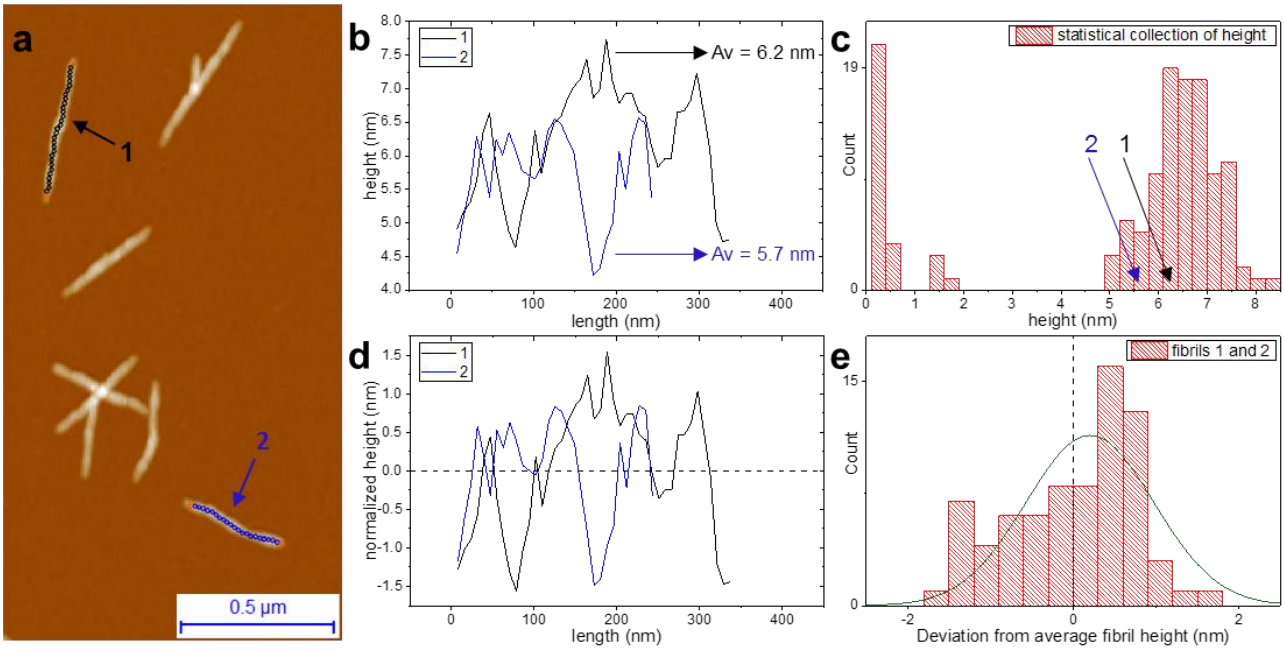

Supplementary Fig. 4. a) Example of the tracing of height and length of fibrillar structures, indicated with 1 and 2. b) Graph with the sections of the traced fibrils and their average height. c) Example of a histogram showing the average height of fibrillar structures. d) Graph with normalized profile of the traced fibrils 1 and 2. e) Histogram distribution of the normalized profile height points.

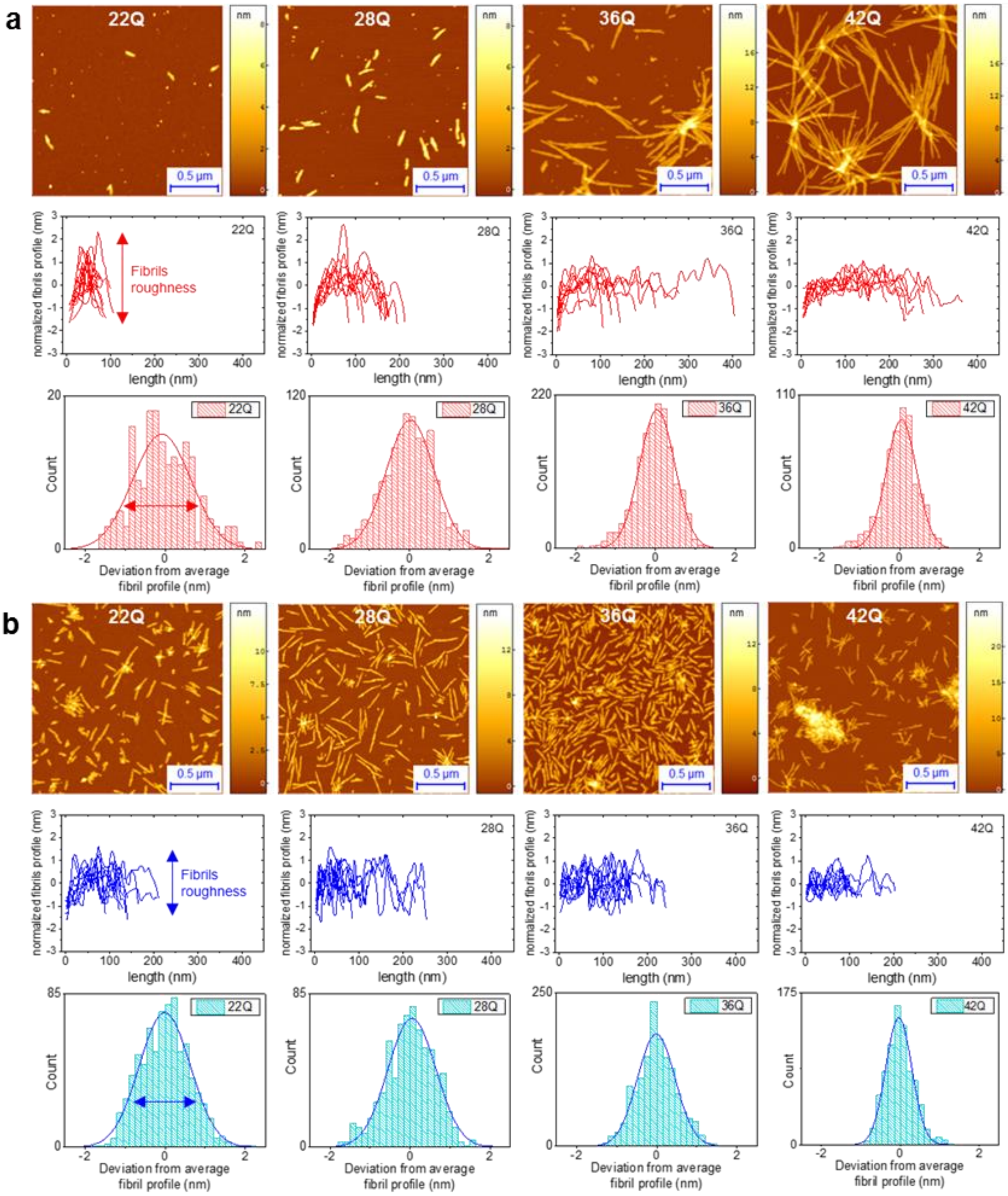

Supplementary Fig. 5. Analysis of fibrils roughness as function of the polyQ content on mica. AFM maps, roughness profiles of fibrillar structures and histograms distribution from average fibril height as function of polyQ content for a) Nt17-truncated and b) full length Exon1 proteins.

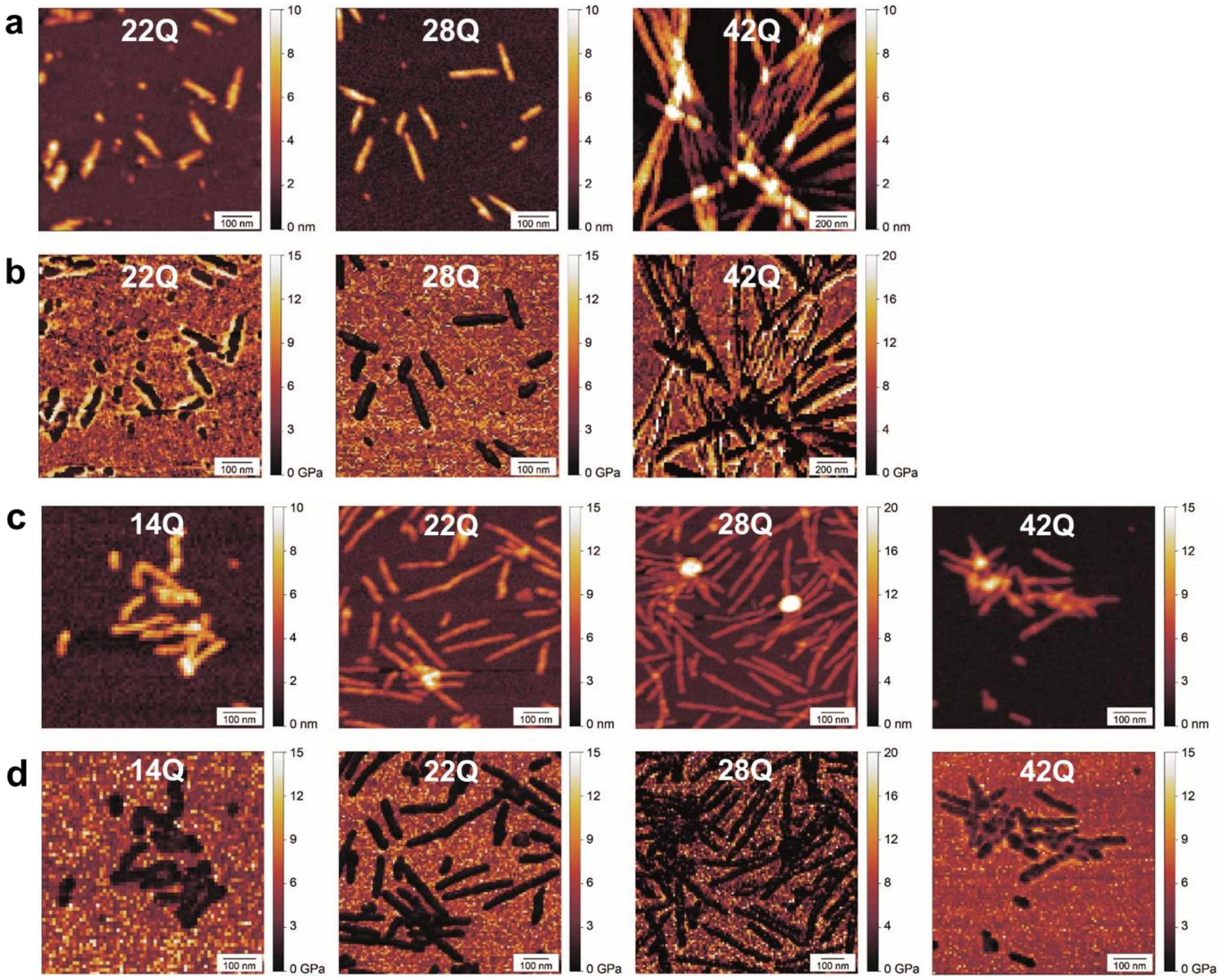

Supplementary Fig. 6. Comparison of morphology and stiffness maps of Exon1 Nt17-truncated Exon1 fibrils. Morphology (a) and stiffness maps (b) of Nt17-truncated Exon1 with polyQ-lengths of14Q, 22Q, 28Q, 42Q. Morphology (c) and stiffness maps (d) of Exon1 with polyQ-lengths of 14Q, 22Q, 28Q, 42Q.

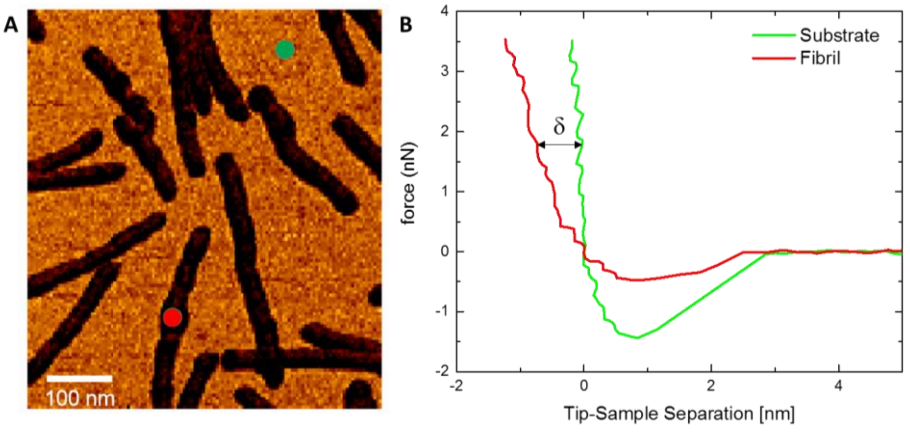

Supplementary Fig. 7. Typical Force Curves on Protein aggregates. A) Stiffness map. B) Single force curves on a fibrillar aggregate (red) and on the substrate (green). δ indicates the indentation depth.

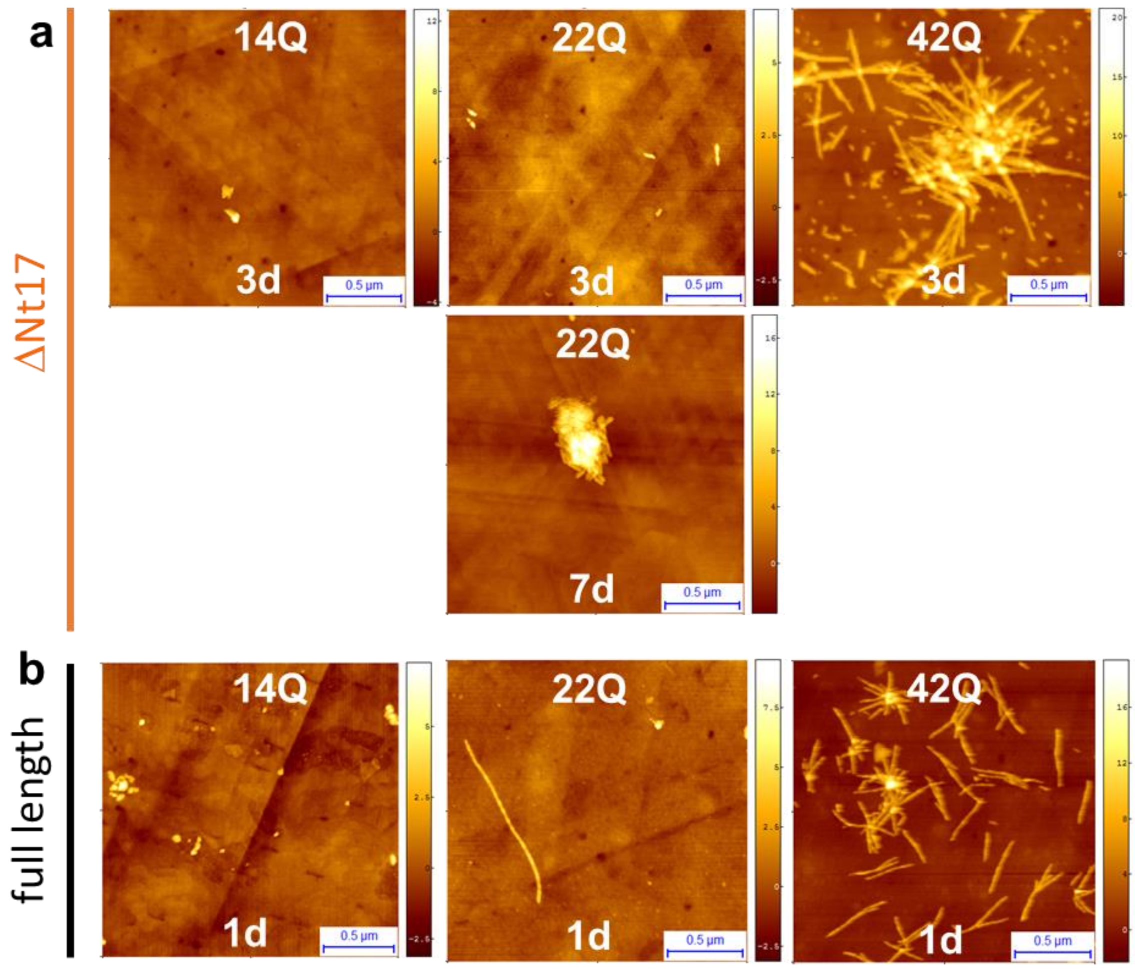

Supplementary Fig. 8. Deposition on gold-coated surfaces. a) Nt17-truncated Exon1 samples after 3 and 7 days incubation and b) Exon1 samples after 1 day incubation with 14Q, 22Q and 42Q.

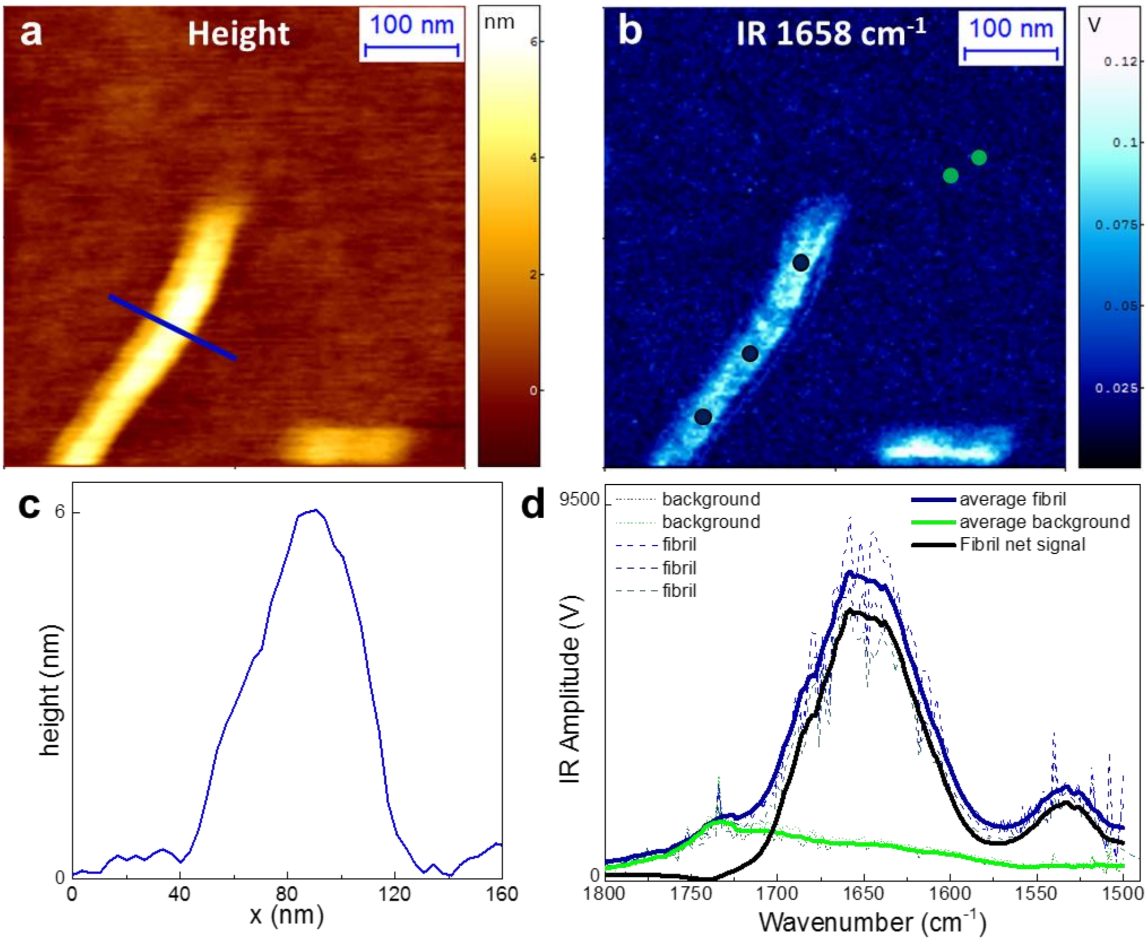

Supplementary Fig. 9. Exon1 42Q single fibril infrared nanospectroscopy. a) AFM morphology map. b) IR absorption map at the QCL resonance peak at 1658 cm^{-1}. c) Cross sectional dimensions of the fibril height. d) IR spectra on different positions of the fibrillar structure (marked in (b) with blue circles) and the substrate (marked in (b) with green circles). The average net signal deriving from the amyloidogenic structure (solid black line) was obtained by subtracting the averaged background signal (solid green line) from the averaged fibril signal (solid blue line).

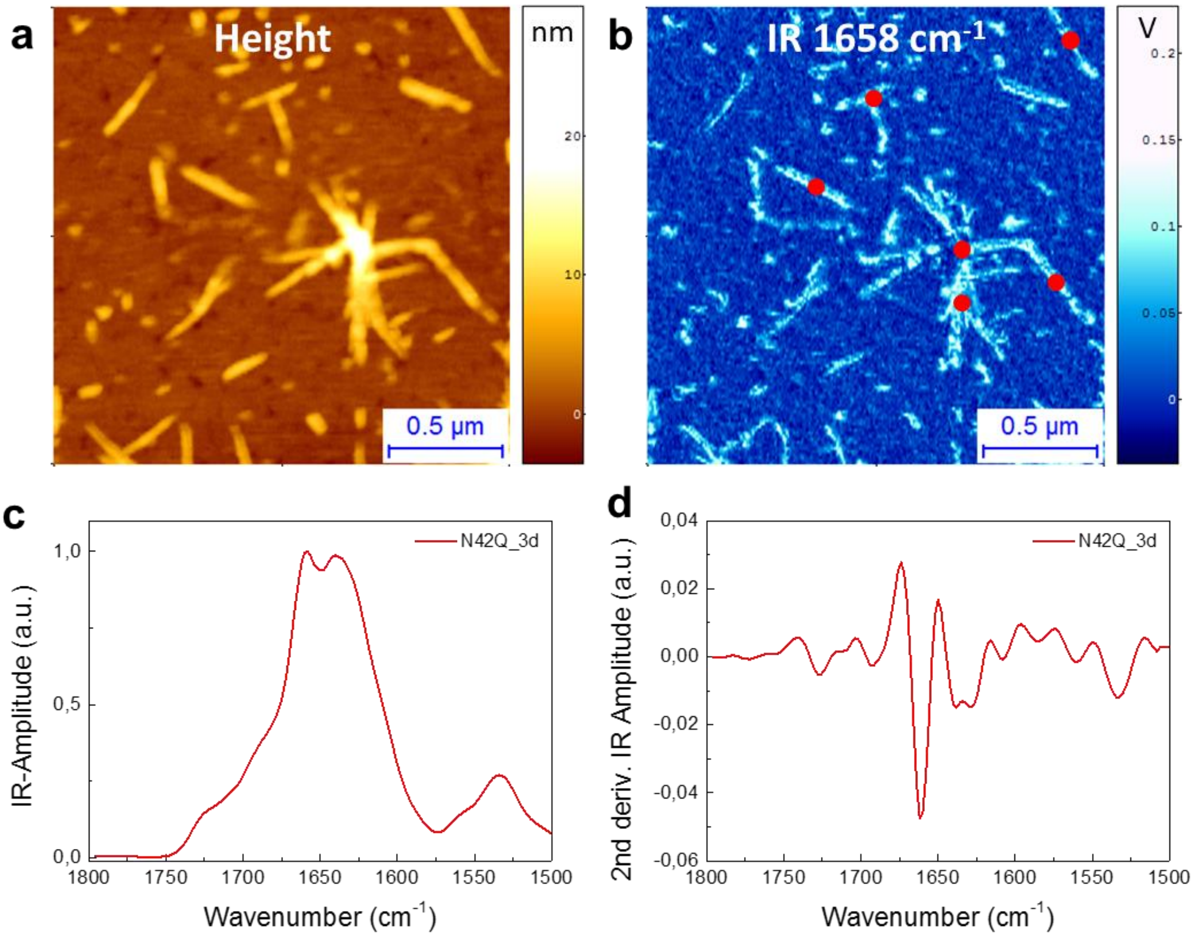

Supplementary Fig. 10. Measurement of IR spectrum of several fibrillar species (Nt17-truncated with 42Q). a) Morphology map. b) IR absorption map in the amide band I at 1658 cm^{-1}. c) Average IR absorption representative points from where the spectra were collected (for each fibril marked with a red circle (b) at least 5 spectra were collected). d) Second derivative of the IR spectrum.

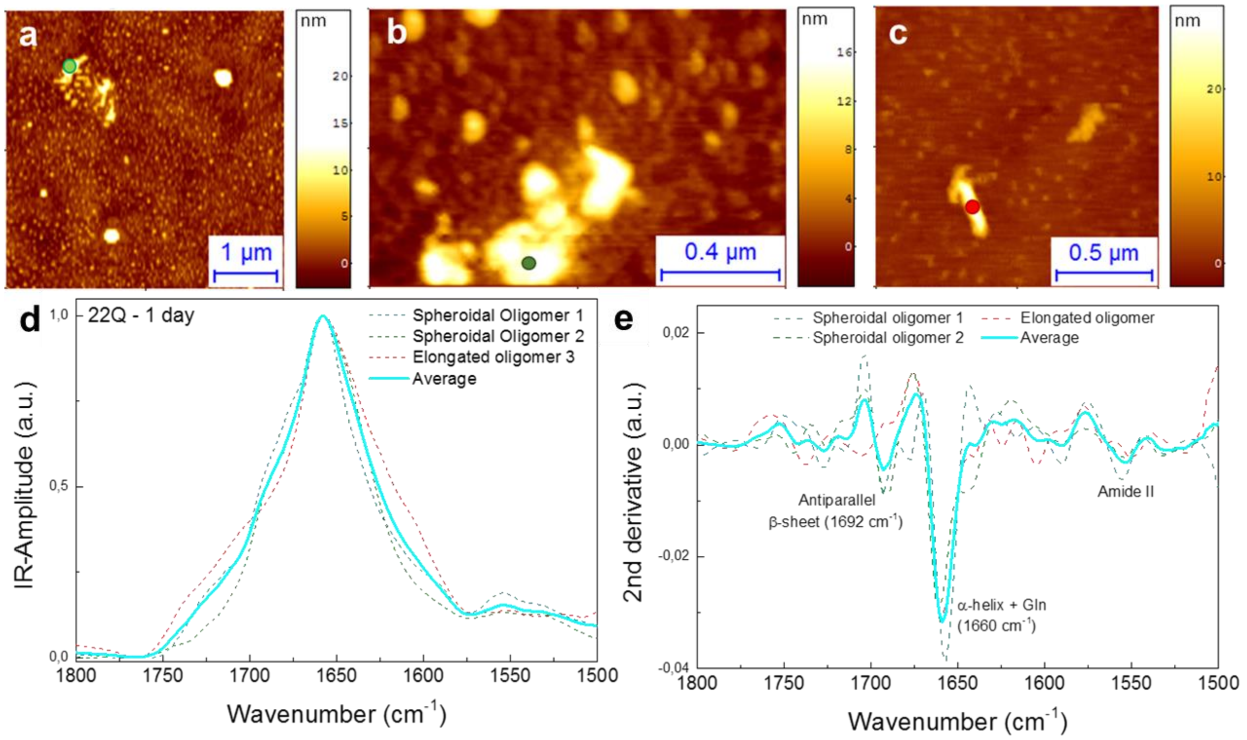

Supplementary Fig. 11. Infrared nanospectroscopy of oligomeric structures of full-length 22Q Exon1. AFM morphology maps of spheroidal (a, b) and elongated oligomers (c). d) IR absorption spectra of spheroidal oligomers (marked in (a, b) with green circle) and elongated oligomers (marked in (c) with red circle) and the averaged spectrum of oligomers (solid blue line). d) Second derivatives of IR absorption and secondary structures components of spheroidal oligomers (blue and green dashed line) and elongated oligomers (red dashed line) are represented together with the averaged oligomeric signal (blue solid line).
